# Artificial intelligence in oncology: Current status and possibilities (Review)

**DOI:** 10.3892/mi.2026.304

**Published:** 2026-02-19

**Authors:** Abhavya Roy, Apurva Bhoyar, Ashok Ahirwar, Yogesh Pawade, Nilesh Chandra

**Affiliations:** 1 University College of Medical Sciences, Guru Teg Bahadur Hospital, Delhi 110095, India; 2 Department of Biochemistry, All India Institute of Medical Sciences, Nagpur, Maharashtra 441108, India; 3 Department of Laboratory Medicine, All India Institute of Medical Sciences, New Delhi 110029, India; 4 Indian Council of Medical Research, Ansari Nagar, New Delhi 110029, India

**Keywords:** oncology, machine learning, artificial intelligence, deep learning, radiomics, predictive oncology, cancer imaging, personalized cancer treatment, digital pathology

## Abstract

Artificial intelligence (AI) is increasingly reshaping oncology by enhancing diagnostic accuracy, improving prognostication and enabling personalized treatment planning. The present review aimed to critically synthesize the contemporary landscape of AI applications across cancer imaging, digital pathology, clinical outcome prediction, chemotherapy and radiotherapy. Recent advances in machine learning and deep learning, particularly convolutional neural networks and transformer-based architectures, have demonstrated robust performance in lesion detection, tumour grading, survival prediction and treatment optimization, in several instances approaching or exceeding expert-level accuracy. Despite these advances, translation into routine clinical practice remains limited due to dataset bias, limited generalizability, the lack of standardized data protocols, insufficient interpretability and regulatory barriers. Ethical challenges related to fairness, transparency and equitable access are especially relevant in low- and middle-income countries. Emerging frontiers, including multimodal AI, foundation models, federated learning, and explainable AI, provide potential solutions to these challenges. Multidisciplinary collaboration, rigorous prospective validation and robust ethical governance will be essential to realize the full potential of AI in advancing precision oncology and improving global cancer outcomes.

## 1. Introduction

Cancer remains a leading cause of morbidity and mortality worldwide, accounting for almost ten million deaths annually and imposing a growing burden on healthcare systems. Modern oncology requires the integration of complex diagnostic, prognostic and therapeutic information derived from medical imaging, histopathology, molecular profiling and longitudinal clinical data. Conventional workflows rely heavily on human interpretation, which is inherently subjective and prone to inter- and intra-observer variability, cognitive bias and information overload. These limitations contribute to variability in diagnosis, risk stratification and treatment selection.

Artificial intelligence (AI), defined as computational systems capable of learning, reasoning and pattern recognition, provides a paradigm shift by enabling automated, objective and data-driven clinical decision support (1). Advances in machine learning (ML) and deep learning (DL), particularly convolutional neural networks (CNNs), have driven major breakthroughs in medical image analysis and predictive modelling (2,3). Unlike early rule-based systems, contemporary AI models can extract high-dimensional features from complex data and model nonlinear relationships that are difficult to capture using traditional statistical approaches.

Multiple systematic reviews and meta-analyses have demonstrated that AI systems can achieve diagnostic performance comparable to, and in some contexts exceeding, that of healthcare professionals in selected oncologic tasks (4-7). Despite this promise, widespread clinical adoption remains limited. Barriers include dataset bias, limited external generalizability, the lack of standardized data acquisition and annotation protocols, insufficient interpretability of model outputs and complex regulatory pathways (8). In addition, concerns regarding data privacy, accountability and equity have become increasingly prominent (9).

The present review aimed to provide a critical, clinically oriented synthesis of AI applications across the oncology continuum. Rather than providing a purely descriptive overview, the present review aimed to discuss the methodological strengths and limitations, unmet clinical needs, and emerging technological paradigms, with the aim of providing a practical roadmap for responsible and equitable integration of AI into oncology practice.

## 2. AI in cancer imaging

Medical imaging represents the most mature and clinically advanced domain of AI applications in oncology. AI has been applied across the entire imaging pipeline, including acquisition, reconstruction, segmentation, detection, classification and prognostication, spanning modalities such as computed tomography (CT), magnetic resonance imaging (MRI), positron emission tomography, mammography and ultrasound.

DL-based reconstruction algorithms have enabled substantial improvements in image quality by reducing noise and artefacts, facilitating lower radiation doses and shorter acquisition times without compromising diagnostic accuracy. These advances are particularly relevant in CT and MRI, where dose reduction and accelerated imaging have direct implications for patient safety and workflow efficiency. AI-driven image reconstruction has also enabled improved visualization of subtle lesions, potentially enhancing early cancer detection (10).

CNN-based computer-aided diagnosis (CADx) systems analyse lesion morphology, texture, and spatial context to support malignancy detection, staging and risk stratification (11). Robust performance has been demonstrated in melanoma classification (12), real-time colorectal polyp detection during colonoscopy (13), the identification of nodal metastases in head and neck cancer (14) and breast cancer risk prediction using mammography (15). Several of these systems have progressed to regulatory approval, reflecting the increasing acceptance of AI-enabled imaging tools by agencies, such as the US Food and Drug Administration (16).

Beyond detection, AI-based imaging facilitates tumour phenotyping and prognostication by integrating information across multiple modalities (Table I). Radiomic and DL features extracted from imaging data have been associated with molecular subtypes, treatment response, and survival outcomes (17). However, a number of published studies remain retrospective and single-centre, with limited demographic and technical diversity, increasing the risk of overfitting and dataset bias (18-20). Variability in scanner hardware, acquisition protocols and reconstruction parameters further constrains generalizability (Table II) (21).

Another persistent challenge is interpretability. The black-box nature of DL models complicates the understanding of decision pathways and limits clinician trust. Explainable AI techniques, including saliency mapping and concept-based models, are increasingly explored to address this limitation (22). Federated learning offers a promising strategy for multi-institutional model development while preserving data privacy (Table II) (23). Future research is required to prioritize prospective, multi-centre validation studies, standardized benchmarking datasets and transparent reporting to enable meaningful clinical translation.

## 3. AI in digital pathology

The digitization of histopathology through whole-slide imaging has catalysed rapid growth in AI-driven computational pathology (Table I). Digital slides enable large-scale analysis using DL architectures such as CNNs, fully convolutional networks, and, more recently, vision transformers. These models have demonstrated high accuracy in tumour detection, grading, and prognostication across multiple cancer types (Table II) (24).

Weakly supervised learning approaches have been particularly impactful, enabling model training using slide-level labels rather than exhaustive pixel-level annotations. Clinical-grade performance has been reported for tasks, such as Gleason grading in prostate cancer and lymph node metastasis detection (25). Beyond morphological assessment, AI models can infer genomic alterations and clinically actionable mutations directly from routine haematoxylin and eosin-stained slides, including in lung and gastrointestinal cancers (26,27).

Several studies have demonstrated that AI-derived histopathological features are associated with survival outcomes and therapeutic response, particularly when integrated with molecular and clinical data. These findings suggest that AI-enabled pathology may serve not only as a diagnostic adjunct, but also as a prognostic and predictive tool. In resource-limited settings, the ability to infer molecular information from routine histology has potential implications for cost-effective precision oncology (26).

Despite these advances, digital pathology faces substantial challenges. Variability in staining protocols, scanner technologies and laboratory workflows limits reproducibility across institutions. Dataset bias and relatively small sample sizes further restrict external validity (28). Regulatory approval is complicated by limited interpretability and the paucity of prospective validation studies.

**Figure 1:**
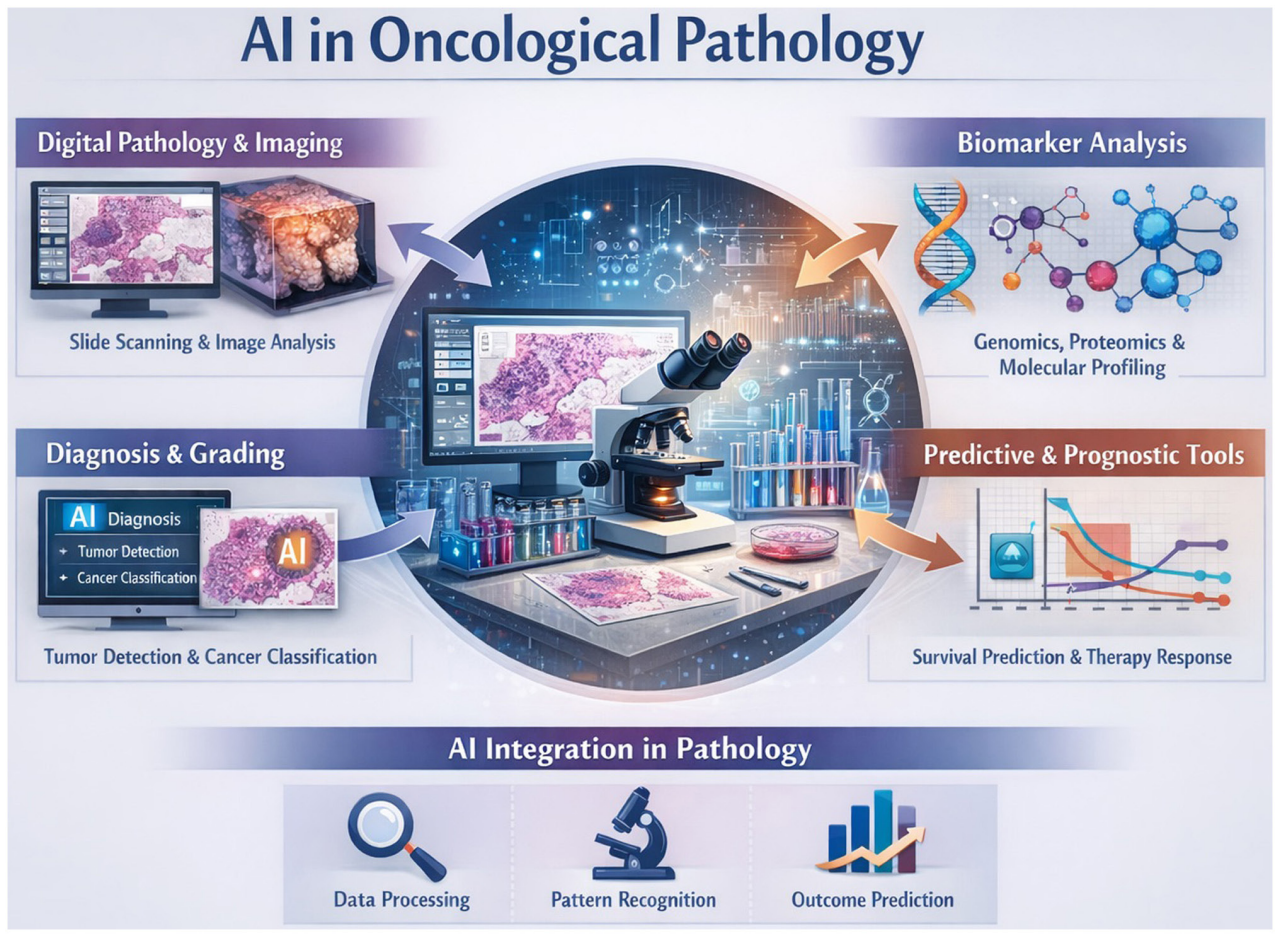
AI in oncological pathology: AI integration across digital pathology, biomarker analysis, tumour diagnosis and grading, and predictive prognostication, highlighting how multimodal data processing supports pattern recognition and outcome prediction to enable precision oncology. AI, artificial intelligence.

Future efforts should focus on standardized pre-processing pipelines, harmonized data annotation practices and pathology-specific reporting guidelines, including extensions of frameworks, such as TRIPOD (29). Explainable AI methods that highlight diagnostically relevant histological features may improve transparency and pathologist confidence. Federated learning frameworks can facilitate multi-centre collaboration while preserving patient privacy, and multimodal integration with radiologic and molecular data may further enhance predictive performance (Table II). The integration of AI across oncological pathology, highlighting its role in digital slide analysis, biomarker profiling, tumour detection and predictive prognostication is illustrated in Fig. 1. The schematic illustration in Fig. 1 emphasizes multimodal data processing from histopathology and molecular features to outcome prediction, demonstrating how AI supports diagnosis, grading and personalized therapy planning within a unified pathology workflow.

## 4. AI in predicting clinical outcomes

Predicting clinical outcomes, such as survival, treatment toxicity and therapeutic response is central to personalized oncology (Table I). AI models integrating radiologic, histopathologic, genomic and clinical data have shown promise in improving risk stratification beyond traditional staging systems (30-33). Ensemble ML methods and deep neural networks have been applied to predict progression-free and overall survival in lung, colorectal, breast and head and neck cancers (34-36).

These models provide several potential advantages, including the early identification of high-risk patients, the proactive modification of treatment strategies and the improved selection of therapeutic modalities. However, a number of outcome prediction models are developed using limited or single-institution datasets, increasing susceptibility to overfitting and reducing external validity (37-40). Interpretability remains a major challenge, particularly when models generate probabilistic outputs without clear clinical actionability.

Another limitation is the limited incorporation of longitudinal data. Cancer progression and treatment response are dynamic processes, and static baseline models may fail to capture temporal changes. Recent research has emphasized the importance of longitudinal modelling using real-world data and post-deployment auditing to ensure safety and performance stability over time (4,41,42) (Table III). Prospective, multi-centre validation and close collaboration between clinicians and data scientists are essential to align AI tools with real clinical needs.

## 5. AI in chemotherapy

Chemotherapy selection and dosing require careful consideration of tumour biology, patient characteristics and the risk of toxicity (Table I). ML and DL approaches have improved the prediction of the drug response by modelling complex pharmacogenomic and molecular interactions that are difficult to capture using conventional statistical methods (43-47). Models trained on large cancer cell-line datasets and multi-omics data have demonstrated superior performance in predicting drug sensitivity and resistance (48).

Despite encouraging preclinical and retrospective results, translation to routine clinical care remains limited. High-quality, well-annotated clinical pharmacogenomic datasets are limited, and the majority of models lack prospective validation (1,49). Limited interpretability further constrains clinician confidence and shared decision-making (Table II) (50,51). Reporting guidelines for clinical trials evaluating AI interventions underscore the need for rigorous study design and transparency.

Future directions include the integration of real-world clinical data, the development of explainable AI frameworks and prospective clinical trials evaluating AI-guided chemotherapy strategies. Reinforcement learning approaches may enable adaptive treatment optimization based on individual patient response trajectories.

## 6. AI in radiotherapy

Radiotherapy is a highly data-intensive discipline, making it particularly amenable to AI-driven optimization. Applications include automated contouring, dose calculation, treatment planning, toxicity prediction, and adaptive radiotherapy (Table I). While Monte Carlo simulations remain the gold standard for dose calculation, they are computationally intensive (52-54). DL models can generate accurate dose distributions rapidly, improving workflow efficiency (55).

Reinforcement learning has been explored for adaptive radiotherapy, enabling treatment plans to evolve in response to anatomical and biological changes during the course of therapy (56). Generative adversarial networks have also been investigated for synthetic data generation to address limited sample sizes and class imbalance. However, challenges related to interpretability, regulatory approval and integration into existing planning systems persist (Table I) (57).

Future research is warranted to prioritize transparent and interpretable models that provide clinically meaningful rationales for dose modification and toxicity prediction. Standardized validation protocols, prospective clinical evaluation, and integration with clinical decision-support systems will be critical for safe and effective deployment.

## 7. Challenges and ethical considerations

The clinical translation of AI in oncology is hampered by regulatory requirements demanding robust evidence of safety, efficacy and generalizability. Data-related challenges (Table III) include inconsistent acquisition, annotation and pre-processing protocols, as well as sampling and observation bias. Models trained on unrepresentative datasets may perform poorly in underrepresented populations, exacerbating health disparities, particularly in low- and middle-income countries (58-60).

Several AI systems in oncology have indeed been withdrawn or scaled back following deployment due to inadequate validation and poor real-world performance. Perhaps the most notable example is IBM Watson for Oncology, which was marketed as an AI-driven treatment recommendation tool, but lacked robust clinical validation; it produced recommendations that often did not align with real-world practice and was ultimately discontinued when hospitals withdrew and IBM sold its Watson Health division after failing to demonstrate clinical utility and safety (61,62). Other older oncology decision support prototypes, including rule-based systems such as OncoDoc and its successors, were never widely adopted outside research settings due to high rates of guideline-discordant recommendations and insufficient validation on diverse clinical data (63). These cases underscore the risks of implementing AI in oncology without rigorous external validation and prospective outcome evaluation before broad clinical use.

Ethical governance frameworks need to prioritize fairness, transparency, accountability and inclusivity to prevent AI from reinforcing existing inequities (Table III). Robust data governance policies are required to balance innovation with privacy, informed consent and security. The engagement of all stakeholders, including patients, clinicians, developers, regulators and ethicists, is essential, as is continuous performance monitoring and post-deployment auditing.

## 8. Emerging frontiers

Several emerging paradigms have the potential to overcome persistent scientific, clinical and implementation barriers in AI-enabled oncology. Among these, multimodal AI (Table II) represents a critical advancement by integrating heterogeneous data sources, namely radiological imaging, digital pathology, genomics, proteomics, laboratory parameters and longitudinal electronic health records into unified predictive frameworks. Such models better capture tumour heterogeneity, temporal disease evolution and patient-specific context, thereby enabling more accurate diagnosis, risk stratification, treatment selection and outcome prediction compared with unimodal approaches (64).

Foundation models (Table II) pretrained on large, diverse and multi-institutional datasets are increasingly influential in oncology. These models leverage self-supervised or weakly supervised learning to acquire generalizable representations that can be efficiently fine-tuned for specific cancer types, modalities, or clinical tasks. Foundation models reduce the dependence on extensive labelled datasets, enhance robustness across populations and scanners, and facilitate rapid deployment in resource-variable settings, including smaller centres (65-67).

Federated learning (Table II) provides a pragmatic solution to data-sharing constraints by enabling collaborative model training across institutions without centralized transfer of sensitive patient data. This paradigm is particularly valuable in oncology, where data scarcity, privacy regulations, and institutional silos limit model generalizability. Federated approaches can improve performance across diverse demographic and clinical settings while maintaining compliance with data protection frameworks (Table II) (68,69).

Despite these technical advances, explainable AI remains central to clinical adoption, regulatory approval and medicolegal accountability. Transparent models that provide interpretable features, uncertainty estimates, and clinically meaningful visualizations foster clinician trust and support safe integration into decision-making workflows (Table II) (70,71).

Finally, ethical and equity considerations are especially salient in low- and middle-income countries. Challenges related to infrastructure, data quality, workforce training and algorithmic bias (Table III) need to be addressed through targeted capacity building, inclusive dataset development and international collaboration. Without deliberate governance and context-aware implementation, AI risks exacerbating existing disparities (72,73). Ensuring fairness, transparency and accessibility will be essential for realizing the global promise of AI-driven oncology.

## 9. Conclusion

AI holds transformative potential to advance precision oncology by improving diagnostic accuracy, prognostication and treatment optimization. Realizing this potential requires rigorous prospective validation, transparent model design, multidisciplinary collaboration and robust ethical safeguards. With responsible development and implementation, AI can become an integral component of oncology practice and contribute meaningfully to improved cancer outcomes worldwide.

## Figures and Tables

**Table I:** AI in clinical fields: Applications and limitations.

Clinical field	Key AI applications	Representative outcomes	Major limitations
Cancer imaging (CT, MRI, PET, mammography, USG)	Lesion detection, segmentation, staging, radiomics-based prognostication, image reconstruction	Improved detection of melanoma, breast cancer risk prediction, colorectal polyp identification, nodal metastasis classification	Retrospective single-centre datasets, scanner/protocol variability, limited interpretability, dataset bias
Digital pathology	Tumour detection and grading, lymph node metastasis identification, genomic mutation inference from H&E slides, survival prediction	Gleason grading, gastric/colonic tumour classification, prediction of actionable mutations	Staining and scanner heterogeneity, lack of standardization, insufficient prospective validation
Clinical outcome prediction	Survival prediction, toxicity risk estimation, response assessment	Enhanced risk stratification beyond conventional staging	Overfitting, poor external generalizability, limited longitudinal modelling
Chemotherapy	Drug response prediction, pharmacogenomic modelling, resistance detection	Improved *in silico* prediction of sensitivity using multi-omics data	Scarcity of high-quality clinical datasets, limited explainability, lack of prospective trials
Radiotherapy	Automated contouring, dose calculation, adaptive planning, toxicity prediction	More rapid treatment planning and adaptive workflows	Regulatory hurdles, integration challenges, black-box models

The information presented in the table is derived from previous studies (17,21-24,30-41,52-54,57,68,69). CT, computed tomography; MRI, magnetic resonance imaging; PET, positron emission tomography; USG, ultrasound sonography.

**Table II:** AI methodologies in oncology: Applications and challenges.

Methodology	Typical applications	Strengths	Challenges
Machine learning (ML)	Survival prediction, drug response modelling, radiomics	Handles structured clinical and molecular data	Feature engineering dependence, limited scalability
Deep learning (CNNs, transformers)	Imaging analysis, digital pathology, outcome prediction	Automatic feature extraction, high diagnostic accuracy	Poor interpretability, large data requirements
Radiomics	Tumour phenotyping, prognostication	Quantifies imaging heterogeneity	Sensitive to acquisition variability
Multimodal AI	Integration of imaging, pathology, genomics, EHRs	Captures tumour biology and patient context comprehensively	Complex model design, data harmonization
Foundation models	Transfer learning across cancers and institutions	Reduced labelling needs, improved robustness	Computationally intensive, transparency concerns
Federated learning	Multi-centre model training without data sharing	Preserves privacy, improves generalizability	Infrastructure demands, communication overhead
Explainable AI (XAI)	Clinical decision support transparency	Builds clinician trust	Often adds complexity, limited standardization

The information presented in the table is derived from previous studies (4,21-29,41-43,47,52-57,64-73). AI, artificial intelligence; CNNs, convolutional neural networks; EHRs, electronic health records.

**Table III:** AI-driven challenges in oncology: Computational, ethical/regulatory and data quality domains.

Domain	Key challenges
Computational	High hardware requirements, model instability over time, lack of standardized benchmarking, workflow integration difficulties
Ethical/regulatory	Limited transparency, accountability concerns, unclear liability, bias amplification, delayed regulatory approval, failures such as IBM Watson for Oncology due to inadequate validation
Data quality	Dataset bias, small sample sizes, inconsistent acquisition and annotation, missing longitudinal data, underrepresentation of LMIC populations
Equity (LMICs)	Infrastructure gaps, workforce shortages, limited digitization, algorithmic bias, restricted access to validated AI tools

The information presented in the table is derived from previous studies (4,23,28,41,42,52-54,58-63,72,73). AI, artificial intelligence; LMICs, low- and middle-income countries.

## Data Availability

Not applicable.
